# Multifrequency Force Microscopy of Helical Protein Assembly on a Virus

**DOI:** 10.1038/srep21899

**Published:** 2016-02-26

**Authors:** Annalisa Calò, Aitziber Eleta-Lopez, Pablo Stoliar, David De Sancho, Sergio Santos, Albert Verdaguer, Alexander M. Bittner

**Affiliations:** 1 CIC nanoGUNE, Tolosa Hiribidea 76, E-20018 San Sebastián-Donostia, Spain; 2 Ikerbasque, Basque Foundation for Science, Maria Diaz de Haro 3, 48013 Bilbao, Spain; 3 Institute Center for Energy (iEnergy), Masdar Institute of Science and Technology, Abu Dhabi, United Arab Emirates; 4 Catalan Institute of Nanoscience and Nanotechnology (ICN2), CSIC and the Barcelona Institute of Science and Technology, Campus UAB, Bellaterra, 08193 Barcelona, Spain

## Abstract

High-resolution microscopy techniques have been extensively used to investigate the structure of soft, biological matter at the nanoscale, from very thin membranes to small objects, like viruses. Electron microscopy techniques allow for obtaining extraordinary resolution by averaging signals from multiple identical structures. In contrast, atomic force microscopy (AFM) collects data from single entities. Here, it is possible to finely modulate the interaction with the samples, in order to be sensitive to their top surface, avoiding mechanical deformations. However, most biological surfaces are highly curved, such as fibers or tubes, and ultimate details of their surface are in the vicinity of steep height variations. This limits lateral resolution, even when sharp probes are used. We overcome this problem by using multifrequency force microscopy on a textbook example, the Tobacco Mosaic Virus (TMV). We achieved unprecedented resolution in local maps of amplitude and phase shift of the second excited mode, recorded together with sample topography. Our data, which combine multifrequency imaging and Fourier analysis, confirm the structure deduced from averaging techniques (XRD, cryoEM) for surface features of single virus particles, down to the helical pitch of the coat protein subunits, 2.3 nm. Remarkably, multifrequency AFM images do not require any image postprocessing.

Since the discovery of the Tobacco Mosaic Virus (TMV) and the subsequent birth of virology over a century ago, TMV has become a paradigm of protein quaternary assembly in biology, and even of self-assembly in general. The virion has a single strand of RNA that is protected by a coat, formed by thousands of units of the same protein, organized in a helix (49 subunits of the coat protein form three helix turns). It has been thoroughly characterised by structural biology techniques^1^,^2^,^3^. Due to its extremely large mass (39.6 MDa)^4^, the system is beyond the reach of nuclear magnetic resonance (NMR), which for structure determination is limited to proteins up to hundreds of kDa. However, both X-ray diffraction and microscopy techniques have resolved intimate details in the three dimensional arrangement of the coat protein and RNA in the virion. X-ray fiber diffraction allowed for modelling the virus at Angstrom resolution^1^. Later on, cryo-electron microscopy techniques (cryo-EM) have greatly expanded the understanding of the nature of this 3D assembly^5^. In both cases, the experimental results were augmented by extensive computational modelling, in order to produce final structural models, an approach that is not devoid of ambiguities^6^.

In cryo-EM, for example, “single particle” analysis methods are used to transform the electron density maps, which do not have resolution in the nm range, into 3D structures, where individual proteins are resolved. This method implies atomistic models to be fitted into density maps of individual virions and needs averaging of thousands of maps from tens or even hundreds of particles to reduce noise^7^. While the atomistic models are usually taken from X-ray diffraction data, independent cross-validation methods are nowadays required as an added value for the analysis, in order to give a reliable structural characterization^6^,^8^.

Despite the experimental limitations, cryo-EM and X-ray diffraction give a coherent view of the TMV architecture. TMV is a hollow, 300 nm long rod, with a diameter of 18 nm, an internal channel of 4 nm, and characteristic repeating distances along the tube surface^4^. The helix pitch, i.e. the axial distance between two coat proteins in one helix turn, is 2.3 nm and the length of three helix turns is 6.9 nm^9^. This is a particularly relevant distance, as it implies an integer number of coat proteins is accommodated along the TMV helix. The 6.9 nm distance runs parallel to the virion axis, from the center of coat protein n to the center of coat protein n + 49, while neither the 2.3 nm single helix pitch, nor the double of 4.6 nm, connect protein centers axially. This organization results from the early stages of virus assembly, where planar, double discs made up of 34 coat proteins dislocate giving rise to a helical structure containing 16.33 proteins per turn^10^,^11^,^3^.

Recently, the three helix turn symmetry was detected in a single virion by ultralow energy electron holography in vacuum^12^. It is remarkable that this is in fact the first proof of the accepted model in a single virion, without averaging. Other single particle methods are very scarce. With atomic force microscopy (AFM) single viruses are easily imaged, but small protein aggregates (capsomers) have been resolved only in the case of highly symmetric icosahedral virions of size in the range 50–100 nm^13^,^14^,^15^. In the highest resolution images produced so far on Tobacco Mosaic virions, isolated topographic features were observed and assigned to unresolved helix repeats^16^. The limitations in the resolution in the case of TMV have been mainly attributed to the very small lateral scale, to tip convolution effects, thermal fluctuations or to water condensation phenomena that may introduce instabilities during scanning^17^. Even if it is customary to perform AFM experiments with viruses in aqueous solution^18^,^19^, ambient air is preferred for imaging TMV, due to its high chemical and mechanical stability^20^,^21^, which is a key factor for its survival and its infectivity. A recent AFM study of single TMV particles in air employed image analysis algorithms, inspired by the cryo-EM methodology, with the aim to denoise the AFM topography and disclose periodic features invisible by simple image inspection^22^. Topographic images of individual virions were filtered in a multi-step image processing, and 3D structures derived from X-ray diffraction were used to validate the procedure. Periodic distances in the 5–10 nm range were obtained as a result of this single image analysis and discussed in relation to the expected helix repeats, mainly to the 6.9 nm building block.

In this work we explore advanced modes to improve the spatial resolution of the AFM technique on top of individual TMVs without *any* image processing. The AFM probe is simultaneously excited at two resonant frequencies, corresponding to the first two flexural modes of the cantilever oscillation, a technique called multifrequency AFM^23^. The excitation of multiple frequencies has the potential to enlarge the possibilities of AFM microscopy beyond the standard lateral resolution, as it opens new channels of observation with an increased sensitivity towards minute samples heterogeneities, due to mechanical or compositional variations. While a huge part of the current research in this field has been dedicated to find theories that correlate the AFM observables in multifrequency experiments to material properties, and to decouple signals from different channels^24^, various experimental configurations have already been tested in the field of polymers and materials science^25^,^26^ and in biology^27^. The potential to yield high-resolution surface features of individual, biological objects has to our knowledge not been exploited.

In our experiments, second mode images of the surface of TMV particles were collected and showed typical features, i.e. oriented periodic stripes due to the helical organization of the coat protein subunits. These features were further characterized in terms of their periodic components by means of Fourier analysis. Periodic stripes were absent in standard AFM topography and phase shift images obtained simultaneously with multifrequency experiments and also in independent experiments with single probe excitation.

## Results and Discussion

**Figure 1:**
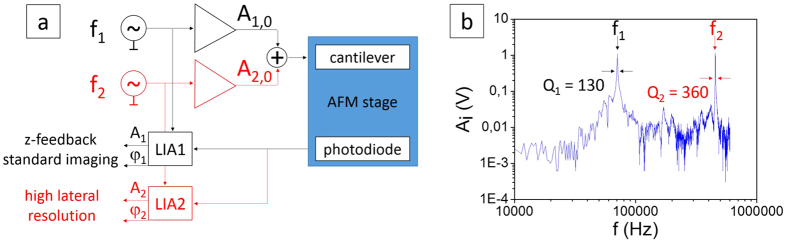
Multifrequency AFM setup. (**a**) Schematics of the configuration of the microscope. A_1,0_ and A_2,0_ are the free amplitudes of the cantilever oscillation at frequencies f_1_ and f_2_, respectively. They can be independently controlled in a multifrequency experiment. (**b**) Spectrum of a Multi75Al cantilever (f_1_ = 75 kHz and f_2_ = 450 kHz, see Methods). The corresponding Q factor of the two modes is calculated from the peaks, as the resonant frequencies divided by the half power bandwidth^30^.

Figure 1a shows the schematics of multifrequency AFM. The cantilever was simultaneously excited at the two lowest resonant frequencies (f_1_, f_2_) by a linear combination of two sinusoidal voltages. In multifrequency AFM, the amplitude of the second excited mode (A_2,0_) is usually set lower than the amplitude of the first one (A_1,0_). In our experiments A_2,0_ = 0.1·A_1,0_^28^. In this way the second excitation can be considered as a perturbation of the first one^25^, allowing for gentle tip-sample interactions, and avoiding tip and/or sample damage. Moreover, we did not see any improvement in the images contrast by varying A_2,0_ around the chosen value, or by using A_2,0_ values below 0.1·A_1,0_.

For our experiments, we deposited TMV on gold substrates, and recorded AFM images, using rectangular cantilevers with standard and super sharp tips (see Methods). Figure 1b presents the resonance spectrum of one of the cantilevers used (nominal tip radius ~10 nm), far from contact with the surface, exhibiting two peaks at f_1_ = 75 kHz and f_2_ = 450 kHz 23,29,30. The signals from the two excited modes were monitored during tip scanning and detected by the microscope ARC2 controller with two lock-in amplifiers (LIAs, see Fig. 1a), separately for f_1_ and f_2_. The amplitude of the first, fundamental, mode (A_1_) is controlled by the feedback loop during scanning, and maintained constant at the set point value to give the sample topography, as in standard amplitude modulation AM-AFM.

### Intermediate resolution of surface features

**Figure 2:**
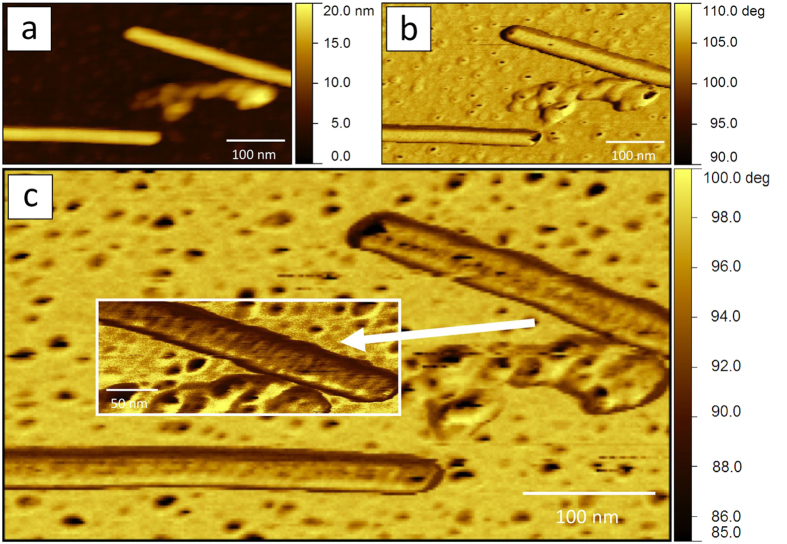
Standard and multifrequency AFM channels (f_1_ = 115 kHz, f_2_ = 720 kHz). Topography of TMV (**a**), phase shift of the first excited mode (φ_1_ image) (**b**), and phase shift of the second excited mode (φ_2_ image) (**c**). Far from contact φ_1_ = φ_2_ = 90°. The scan rate is 1 Hz, images resolution 256 × 142 pixels and the fast scan horizontal. (c, inset) Detail of the non-horizontal virion (φ_2_ image, z scale = 70–114°). Scan rate is 1 Hz, resolution is 512 × 194 pixels and the fast scan horizontal. The topography image (**a**) has been flattened excluding the highest particles region, while images in (**b**, **c**) and (**c**, inset) have not been processed.

Similarly to standard single frequency AFM, data recorded at the lowest resonance frequency f_1_ give access to surface features at intermediate resolution. Figure 2a,b are examples for such standard AFM information, i.e. the topography and the phase shift relative to the drive force oscillating with f_1_ (φ_1_). New information that we obtain simultaneously at f_2_ is the amplitude and the phase shift of the second mode (A_2_ and φ_2_, both recorded in open loop). Figure 2c shows the φ_2_ image of the same sample region in Fig. 2a,b.

Two TMV particles at different orientations are visible in the images presented in Fig. 2. Our sample substrate was not completely flat, due to the presence of protein aggregates, presumably single disks and/or vertically stacked disks of 17 coat proteins, having a diameter of 18 nm. This is a quite usual feature in the case of biological samples^31^. The experimental conditions (scan rate, amplitude set-point and feedback gains) were optimized to have a minimum error signal as seen from the A_1_ image (see Supplementary Fig. S1). Here, contrast is only observed for the largest protrusions, and on the edges of TMV particles, whenever they are not parallel to the fast scan direction (horizontal, throughout the paper).

At the same time, images were collected in the net attractive tip-sample interaction regime, i.e. all φ_1_ values in Fig. 2b are above 90° (see Methods)^32^. We achieved this by applying relatively small free amplitudes A_1,0_ = 10–20 nm^33^, and by choosing amplitude set points as close as possible to the free amplitude, but compatible with high image quality. This was confirmed by an almost ideal overlap in trace and retrace images. Our operational regime is defined in amplitude and phase shift curves, recorded vs. the z-piezo position at f_1_ for the standard cantilever used in this work, as it can be seen in the Supplementary Fig. S2.

In the attractive tip-sample interaction regime, the contact time between the tip and the sample is minimal, while the force exerted on the sample can be kept well below 1 nN (see Supplementary Fig. S2). This allows not only preserving soft biological materials while scanning, but also sustaining high sensitivity to the very surface of the sample. Additionally, it allows tracking lateral variations, roughness, and/or physico-chemical heterogeneities, without mechanical contact and potential sample deformation^32^.

Individual TMV virions exhibit heights of ~14 nm, as determined from the height distribution histograms of topographical images, like the one shown in Fig. 2a. This slight deformation (virions thickness is expected to be 18 nm) is well established for hydrophilic substrates^4^,^34^. Moreover, we clearly find a pattern on the TMV surface in the φ_2_ map (see Fig. 2c and inset, showing a detail of the non-horizontal virion). This is the first time that such a fine regular structure is observed on TMV by AFM without any image processing. Note that φ_2_ maps do not require even the most basic AFM image treatments, such as leveling, removal of aberrant scan lines, or z scale adjustments. This pattern, which is absent in the topography and in the A_1_ and φ_1_ signals, contains parallel stripes of a regular periodicity.

### High resolution features

In multifrequency AFM, an increase in the lateral resolution is expected when other frequencies are excited simultaneously with the first fundamental frequency. This is based on activating channels that are independent from topography recording^23^,^25^. These new channels, namely the amplitude and the phase shift for each new excited mode (A_i_, φ_i_), are expected to be sensitive to very tiny variations of the tip-sample interaction force, in the range of pN, and active at larger separation distances. The increased sensitivity is attributed to the higher Q factor of higher modes (see Fig. 1b)^24^,^35^. Even in the simplest configuration, i.e. where only the first two flexural modes are excited, the signal of φ_2_ can be ten times larger compared to φ_1_^36^. Hence, φ_2_ should give measurable differences even for very similar materials (hydrocarbons of different chain lengths), or for Hamaker constants differing by merely 5%. Remarkably, the attractive interaction regime is the ideal interaction regime to probe such modes and imaging channels^35^.

**Figure 3:**
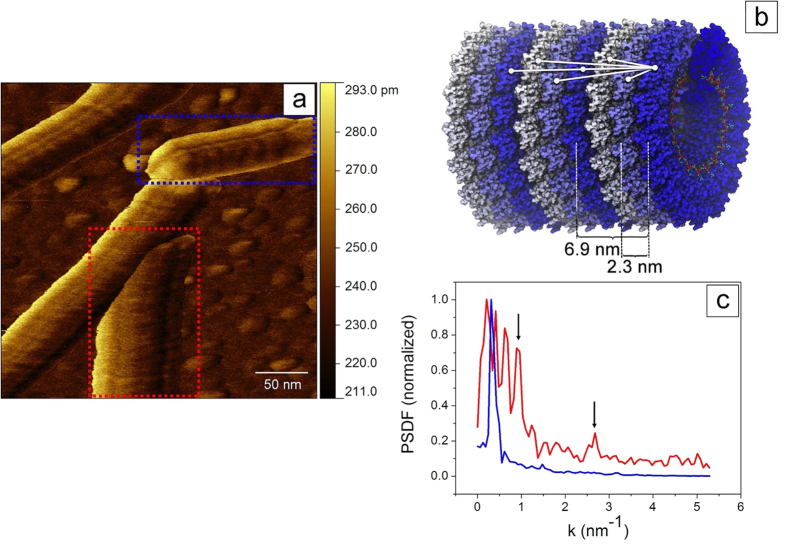
Periodic distances of TMV particles from multifrequency AFM images (f_1_ = 65 kHz, f_2_ = 430 kHz). (**a**) A_2_ image showing TMV particles on gold in various orientations. The topographic height of TMV is 14 nm, the resolution 512 × 512 pixels, the scan rate 1 Hz and the fast scan is horizontal. (**b**) Sketch of the TMV coat protein assembly containing three fundamental axial building blocks. Axial and near-axial distances between protein turns (16.33 proteins) are indicated. The off-axis angles for n turns of the helix are 27°, −14°, 0°, 7°, −6°, and 0° for n = 1… 6. (**c**) Averaged power spectrum density function (PSDF) from a region inside the vertically (red plot) and the horizontally (blue plot) oriented virions in (**a**). These two regions are highlighted in the image in (**a**). The spatial frequencies 0.91 nm^−1^ (6.9 nm) and 2.73 nm^−1^ (2.3 nm) are indicated with black arrows. Here the wavenumber k is equal to 2 π/*l*, *l* being the distance along the main tube axis.

Although our data are apparently at the very limit of lateral resolution, a detailed analysis reveals further information that is not available from standard AFM. For this, it is common practice to apply advanced image processing to visualize small features, which are not directly evident from visual inspection^22^,^37^. However, processing is prone to produce artifacts, which can easily lead to misinterpretations. Hence in the following we analyze the full Fourier power spectrum (averaged power spectrum density function, PSDF, see Methods), *without* any modification or filtering of our data (Fig. 3a,c).

First, the analysis provides the axial periodicity of the above-mentioned stripes. Stripes were found on all the virions, independent of their orientation. They were neither affected by the scan rate, nor by the scan direction, and they are clearly distinguished from noise. The multifrequency A_2_ map of Fig. 3a contains TMV oriented at various angles with respect to the fast scan direction (horizontal). Here, an almost horizontally and a vertically oriented virion are highlighted in blue and in red, respectively. We always found much richer Fourier spectra for TMV oriented perpendicular to the fast scans (vertical TMV). With increasing alignment of TMV with the fast scan direction, the spectra reduce to a single feature, with a periodicity between 10 and 20 nm. An example is the spectrum in blue in Fig. 3c. Here a single broad peak matches the lateral distance between the dark stripes visible on the TMV surface (~15 nm), corresponding to visual inspection and to image profiles.

In the case of the vertically oriented virion, the red spectrum in Fig. 3c exhibits peaks at 9.2 nm (k = 0.68 nm^−1^ = 2π/9.2 nm^−1^), at 6.9 nm (k = 0.91 nm^−1^), and at 2.3 nm (k = 2.73 nm^−1^). The 6.9 nm periodicity can be recognized by direct inspection and matches the axially symmetric three helix turn^3^,^9^,^12^,^22^. Although the low signal to noise ratio prevents further analysis of the 2.3 nm peak, this distance corresponds to the width of a single helix turn, and thus to the width of a single coat protein.

Our results indicate that the structure of TMV is made up of repeating 6.9 nm building blocks, similar to unit cells in a 1D crystal. In this case, the Fourier spectrum should exhibit a 6.9 nm peak plus a series of harmonics^38^, whose intensities reflect how accurately the tip can resolve the fine structure of the fundamental building block. A scenario of a tip with a very big curvature radius, or conversely of a very compliant material, which is flattened under scanning, would give very low harmonic content. Instead, a scenario where the smallest details of the surface roughness can be detected, i.e. when a very sharp tip is used, or when a highly rigid material is imaged, would provide very high harmonic intensities. According to this, the peak we see at 2.3 nm is a possible harmonic of the 6.9 nm distance, giving information about the fine structure of the fundamental building block (see Fig. 3b). Remarkably, we do not observe the first harmonic at 6.9/2 = 3.45 nm, which does not match any relevant distance in TMV from the point of view of structural biology^10^,^39^. In passing, we note that further harmonics beyond k = 2.73 nm^−1^ can be present, although their appearance is likely to be hindered by the instrumental resolution.

Other distances different than 6.9 nm cannot be considered periodic in the classical sense, as complete single or two helix turns would not lead to the same protein position in the TMV lattice. Nevertheless, it is fascinating that our data fit to various multiples of 2.3 nm, the well-known single helix turn of TMV^1^,^5^. It could be argued that the 6.9 nm peak is very strong because the corresponding distance is exactly axial, ideally suited for an axially oriented PSDF. This situation is likely to be very different from the case of the 4.6 nm distance, where the inter-protein distance vectors are 14° off-axis (see Fig. 3b); such a large value is sufficient to suppress the peak below the noise level. The observed 2.3 nm peak is just above the noise level (the corresponding vector is 27° off-axis, but many more vectors contribute for such a short distance). The 9.2 nm distance vector is merely at 7° off-axis, and indeed gives rise to a strong peak at k = 0.68 nm^−1^. Longer distances fall in the order of the overall length of our integration box, and are represented by very few points in the PSDF, thus not accessible, although they would be all oriented less than 7° with respect to the TMV main axis (see Fig. 3b).

The 2.3 nm periodicity confirms that the multifrequency setup allows following the fine structure of the TMV down to the fundamental building block, i. e. one single turn of the helix, the size of a single virion coat protein. Figure 3a is thus a combination of state of the art high resolution AFM image, with local information (amplitude of the oscillation at a high frequency cantilever mode) that accesses otherwise hidden information on local structure, down to the size of small proteins (Fig. 3b).

### The influence of the scan direction

Focusing on the virion orientation relative to the scan direction (horizontal), we tentatively explain why we obtain many spectral components in multifrequency images of vertical virions, but not of horizontal ones (Fig. 3a,c). Anisotropies in TMV particles, as determined by AFM, have already been observed in standard imaging, depending on the orientation with respect to the fast scan direction. This translates, for example, into a large dispersion in the width of differently oriented virions, which has been related to tip convolution effects^22^ and which we also noticed in our experiments.

It has to be pointed out that, in our experimental conditions, only in the case of horizontal virions we were able to stabilize the attractive regime for both excited modes, and to perfectly maintain the amplitude set-point during imaging. These two facts translate into φ_1_ and φ_2_ values that are always above 90°, and into error signal images without contrast inside the whole virion body (see the Supplementary Figs S1 and S5 available online).

This is different for any non-horizontal virion. In Fig. 3a, when the tip touches the left side of the vertical virion, a clear increase in the amplitude of the second mode is observed. In this region we found φ_1_ > 90°, but φ_2_ < 90°. It seems that, in the presence of multiple excitations, jumps to the repulsive regime are observed even when not expected, due to the small amplitudes employed^32^ (see also Fig. 2c and inset). In these conditions the force, or the gradient in the force applied to the sample, can vary substantially across the virion surface. Although this may cause instabilities^40^, it could also make a wide range of interaction regimes available, while imaging an individual virion, thus benefitting high resolution imaging^23^. Exactly this modulation of the interaction force appears to be especially suitable for the detection of the TMV surface structure.

**Figure 4:**
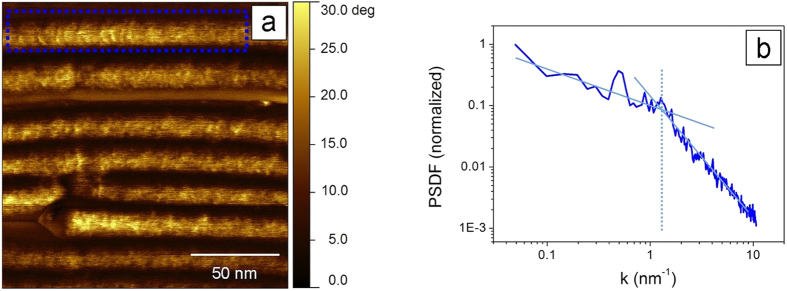
Standard AFM imaging. (**a**) AM-AFM phase image of TMV on mica (topographic height of TMV: 12 nm. For z scale values, see Methods). Image resolution is 520 × 520 pixels and scan rate is 0.7 Hz. (**b**) Averaged PSDF from the region inside the TMV marked in blue in (a). The continuous lines are guides to the eyes; they intersect at k = 1.25 nm^−1^ (*l* = 5 nm). The only observed peak is found at k = 0.5 nm^−1^ (*l* = 12.5 nm).

In order to put our findings in the context of standard high resolution AFM imaging, we compare multifrequency results to monomodal imaging, using an instrument without multimodal capabilities (see Methods; in the Supplementary Information we use also maps from the first excited mode in multifrequency experiments). We optimized standard AFM imaging for having the highest lateral resolution, in terms of imaging parameters (amplitude set-point, scan rate, and number of pixels), probe characteristics (super sharp tips with radius below 6 nm), and sample preparation. For this, we produced TMV surfaces as flat as possible^41^,^42^, containing extended mono- and multilayers of aligned virions. Optimization of imaging was done at the expense of the operational regime. Figure 4a shows the phase image of a monolayer of TMV deposited on mica. The corresponding PSDF, reported in double logarithmic scale, is shown in Fig. 4b. Here and in the linear plot (see Supplementary Fig. S3), we found very few peaks, if any, in contrast to the multifrequency mode.

For a rough surface exhibiting periodic components, peaks are expected in the Fourier spectrum^43^. In the case of rough surfaces, which do not show periodicities, but where characteristic lengths are present, i.e. where a typical average distance is preserved between surface features, one should expect regions of various linear slopes in the double logarithmic plot. The transition region between two slopes defines a correlation length^44^. In this way one can extract, for example, the persistence length of DNA molecules randomly deposited on flat surfaces^44^, or the typical size of islands grown by diffusion limited aggregation^45^.

From the image in Fig. 4a, some surface structure is recognizable inside the virion region, and indeed the Fourier spectrum (Fig. 4b) shows a peak, indicating an axial periodicity of ~12 nm. No other peaks can be identified at higher frequencies. However, we can fit two linear slopes (see Fig. 4b), which intersect at ~ 5 nm. Statistics on 70 individual virions gave a correlation length of (5.5 ± 1.7) nm^22^. Therefore, standard AFM images, even under conditions of high lateral resolution, merely provide a characteristic, non-periodic distance, which comprises the 6.9 nm axial repeat distance in TMV. Possibly, fluctuations or jitter in the phase signal prevent the detection of periodic distances in this case.

Comparison between PSDF obtained in channels from the first and the second excited mode in simultaneous multifrequency experiments are shown in the Supplementary Figs S4 and S5.

## Conclusions

Comparing standard AM-AFM with multifrequency AM-AFM (simultaneous probe excitation with two flexural modes), we find that only the latter can identify the surface organization of Tobacco Mosaic Virus (TMV) at the protein length scale (~ 2 nm). This is possible from fast scans, without the need to access the very limit of resolution. This is evident from a comparison of the imaging conditions for monomodal and multifrequency imaging showed in this work. The Fourier spectra from multifrequency images provide the most detailed fingerprints of the virion architecture, namely the pitch of the virus helix (2.3 nm) and the pitch of three helical turns (6.9 nm). Our experiments show that the multifrequency AFM technique can reveal intimate details of the protein envelope of the virion in a model-free way, even from a single image. Since most biological and biomimetic systems have highly curved surfaces^46^,^47^, we believe that future multifrequency experiments will give new insights in the organization of proteins and other biomolecules in these systems.

Furthermore, our method definitively allows disentangling sample preparation issues from purely instrumental issues that often do not permit to observe details of biological surfaces. It is in fact possible that limitations in AFM imaging might be related to interactions with substrates or to the deposition process affecting the sample “viability”, in the sense of structural integrity. In our example, we show that the true structure is heavily masked in standard AFM, possibly by fluctuations or jitter in the phase signal.

From a general point of view, the multifrequency technique can be extended to other systems where an *a priori* estimation would predict AFM lateral sensitivity being sufficient to resolve surface features, but where such resolution is not obtained in practical cases. Or, conversely, when such sensitivity is hard to achieve due to the high curvature of the surfaces under analysis, in the range of tens of nm. Furthermore, it can shed light on the length scales of the organization of elongated structures, including helical macromolecular assemblies, flagella, and other filamentous viruses.

## Methods

### Sample preparation

Dialyzed TMV solutions in water (40 μL, concentration: 0.05 mg/mL, strain *vulgare*) were incubated on template stripped ultraflat gold surfaces^48^ and let dry during 12 h. Silicon 0.5 × 0.5 cm^2^ surfaces were glued face down on commercial 150 nm annealed gold on mica (SPI Supplies) by means of a mixture of an epoxy glue 1:1 w:w (OptiCLEAR SF850 A&B, Polysciences Inc.). Gold surfaces were obtained after curing (90 minutes, 150 °C) by gently scratching around the glued gold/Silicon pieces with sharp tweezers. The roughness of these substrates, as determined by AFM from 2.5 × 2.5 μm^2^ AM-AFM images, was 0.4 nm. Fresh gold surfaces were cleaned by dipping them first in pure acetone (99.5%, Sigma), then in isopropanol (99.8% Sigma), then in milli-Q water (18 MOhm·cm, <5 ppb TOC), and finally dried. They were made hydrophilic before TMV deposition by an Oxygen plasma for 8 minutes at P = 1 mbar (Standard Plasma System Femto, Diener Electronic GmbH).

For standard high resolution AFM imaging, a drop of 40 μL of dialyzed 1 mg/mL aqueous TMV solution was incubated on freshly cleaved mica, and left drying overnight. TMV concentration and substrate were chosen in order to have extended monolayers of TMV on mica. This reduces height variations due to the close packing of individual virions inside the monolayers and at the same time minimizes virions deformation. Under these conditions, the TMV aspect ratio, i.e. the topographic thickness divided by the width, was the highest (≥0.5)^4^, while higher sample deformations were observed for single virions away from the monolayers, at the TMV-mica interface. High resolution AFM images were taken inside a TMV monolayer (see Fig. 4a). In these conditions, we did not find differences in the topography and in PSDFs of monolayers of horizontal and vertical virions.

Dialysis of TMV solutions was performed by filling dialysis cups (Slide-ALyzer^TM^ 10K MWCO, Thermo Fisher Scientific) with a maximum volume of 250 μL of aqueous TMV solutions (1 mg/mL), and leaving the cups in a beaker filled with 500 mL of milli-Q water, and shaking for 15 min. The water inside the beaker was exchanged six times.

### AFM imaging

Multifrequency AFM experiments were performed with a MFP3D microscope equipped with a cooler/heater sample stage (Asylum Research, Santa Barbara). Measurements were performed in dry conditions, by flowing nitrogen inside the sample holder for 15 min before scanning. In our experimental conditions we did not find evidences of water menisci formation or capillary interactions, for example strong adhesion forces that reflect in the φ_1_ signal, and that could affect the topography, giving rise to unrealistic TMV geometries. We took special attention to the environmental conditions because it is known that TMV surface features can be obscured even by very thin water films^49^. Standard AFM tips mounted on rectangular cantilevers (Multi75Al, nominal f_res_ = f_1_ = 75 kHz, spring constant = 3 N/m and tip radius R < 10 nm, BudgetSensors) and super sharp (SSS-SEIHR, nominal f_res_ = f_1_ = 130 kHz, spring constant = 15 N/m and tip radius R = 2 nm, Nanosensors) were used in experiments, obtaining similar results. The corresponding f_2_ for the two cantilevers used was ≈450 kHz and ≈700 kHz, respectively. 256 or 512 pixels high resolution images were collected at the scan rate of 1–2 Hz.

Standard AFM images were collected simultaneously with the multifrequency maps (see above), but also separately, using an Agilent 5500 microscope equipped with a high resolution scanner and with a chamber for environmental control (Keysight, Santa Clara). The Agilent 5500 is not equipped with multimodal setup. Furthermore, it has to be noticed that in this microscope the phase shift φ is = 0° out of contact, <0° in the attractive regime, and >0° in the repulsive regime. Images were collected at low humidity levels, after flowing nitrogen through the microscope chamber, and monitoring the relative humidity (RH ≤ 10%). High resolution images were obtained collecting 1024 pixel images at a rate ≤0.8 Hz. Tip with nominal radii below 6 nm were used in this case (ACST, nominal f_res_ = f_1_ = 150 kHz, spring constant = 7–8 N/m, AppNano).

Flattening of topographical images was performed using the Igor software implemented in the MFP3D microscope and the Gwyddion software (www.gwyddion.net). Fourier analysis was performed with Gwyddion.

### Fourier analysis

The more straightforward procedure to recognize characteristic distances is to do the Fast Fourier Transform (FFT) of a single scan line. Nevertheless, as usual with bio-systems, we found our scans to be very noisy, and the standard procedure hardly gave any peak above the background. A much more robust method is the averaged PSDF, where many lines are considered. Mathematics and proper definition can be found in http://gwyddion.net/documentation/user-guide-en/statistical-analysis.html. In the following, we comment about the rationale. In PSDF, the FFT of each individual line is computed, then the square norm (the sum of the square of each spectral contribution, which is a purely real value) determined, and finally averaged between many scan lines. In this way, an increase in statistics is obtained at the expense of losing the phase information. This is sufficient in our case since we are only concerned about periodic distances. The analysis presented in Fig. 3 is based on the fully averaged PSDF extracted along the main axis of the TMV. In order to obtain the spectra of the virions in Fig. 3b, boxes in the substrate and in the virion region were compared to confirm that the analyzed peaks were not present in the background. Also, once the peaks were identified, we optimized the box size to maximize the signal to noise ratio. We notice that for such an extraction, particular care must be taken for the experimental conditions. For example, besides working with new tips, we checked that the tip radius was maintained *in situ*, i.e. during experiments^50^. As general rule, we confirmed all the results for the tilted virions in Figs 2c and 3a (see, for an example, Supplementary Fig. S4). Yet, we refrain from further conclusions based on these data to not deal with artifacts introduced by applying rotation matrices. Topographical images were not analyzed by PSDF because the flattening process prevents any access to sample periodicities.

## Additional Information

**How to cite this article**: Calò, A. *et al.* Multifrequency Force Microscopy of Helical Protein Assembly on a Virus. *Sci. Rep.* **6**, 21899; doi: 10.1038/srep21899 (2016).

## Supplementary Material

Supplementary Information
