# Supplementary material for: Multifrequency Force Microscopy of Helical Protein Assembly on a Virus
Source: Sci Rep. 2016 Feb 26;6:21899. doi: 10.1038/srep21899 (PMC4768132; doi:10.1038/srep21899)
Supplement: Supplementary Information [file srep21899-s1.pdf]

Supplementary Information

Multifrequency Force Microscopy of Helical Protein Assembly on a Virus

Annalisa Calò[a*], Aitziber Eleta-Lopez[a], Pablo Stoliar[a], David De Sancho[a,b], Sergio Santos[c], Albert Verdaguer[d], Alexander M. Bittner[a,b]

[a] CIC nanoGUNE, Tolosa Hiribidea 76, E-20018 San Sebastian-Donostia, Spain

[b] Ikerbasque, Basque Foundation for Science, Maria Diaz de Haro 3, 48013 Bilbao, Spain

[c] Institute Center for Energy (iEnergy), Masdar Institute of Science and Technology, Abu Dhabi, United Arab Emirates

[d] Catalan Institute of Nanoscience and Nanotechnology (ICN2), CSIC and the Barcelona Institute of Science and Technology, Campus UAB, Bellaterra, 08193 Barcelona, Spain

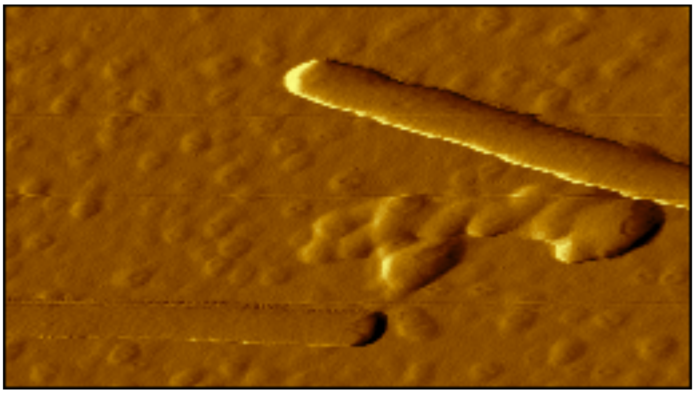

Fig. S1. Standard amplitude (A_1) image (first mode) from the sample shown in Fig. 2 of the manuscript (Z scale = 17-19 nm). Strong contrast is observed only at the edges of non-horizontal TMV virions.

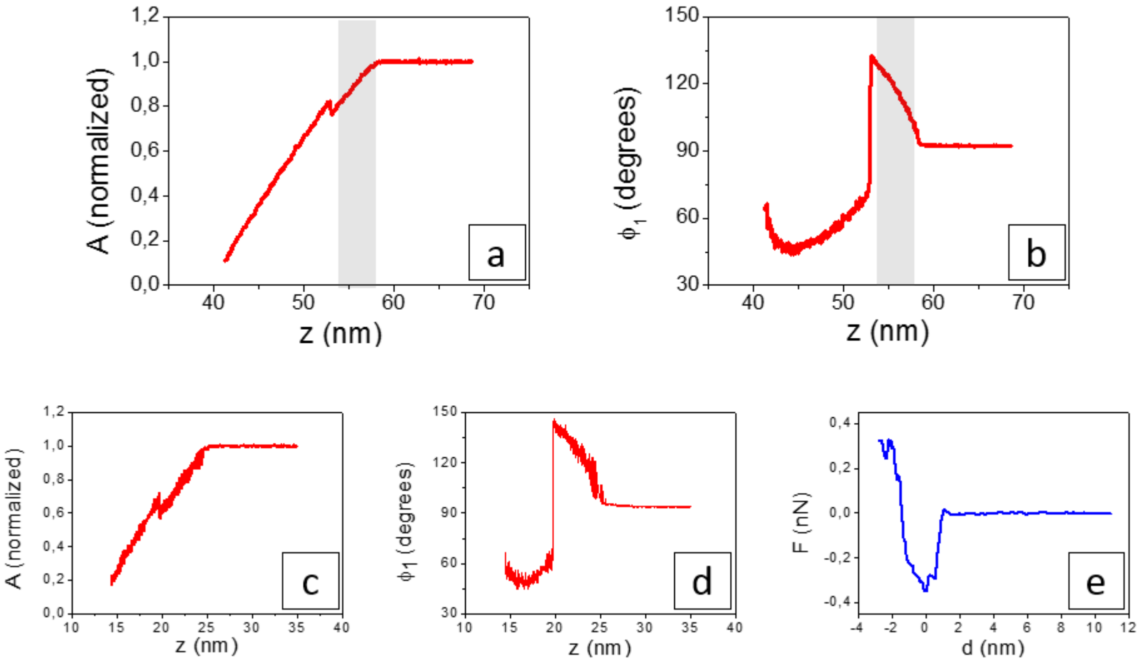

Fig. S2. Attractive tip-sample interaction regime. Amplitude (a) and phase shift (b) vs. z-piezo position (APD curves), taken on gold with a cantilever of resonance frequency f_1 = 75 kHz ($A_{1,0}$ = 20 nm). The attractive operation regime is indicated in grey. (c, d) APD curves ($A_{1,0}$ = 15 nm) collected on top of a TMV particle. (e) Tip-sample

conservative force reconstructed from the APD curves in (c) and (d,) according to the Sader-Jarvis-Katan formalism[1-3]. The peak force (minimum in (e)) is 0.35 nN.

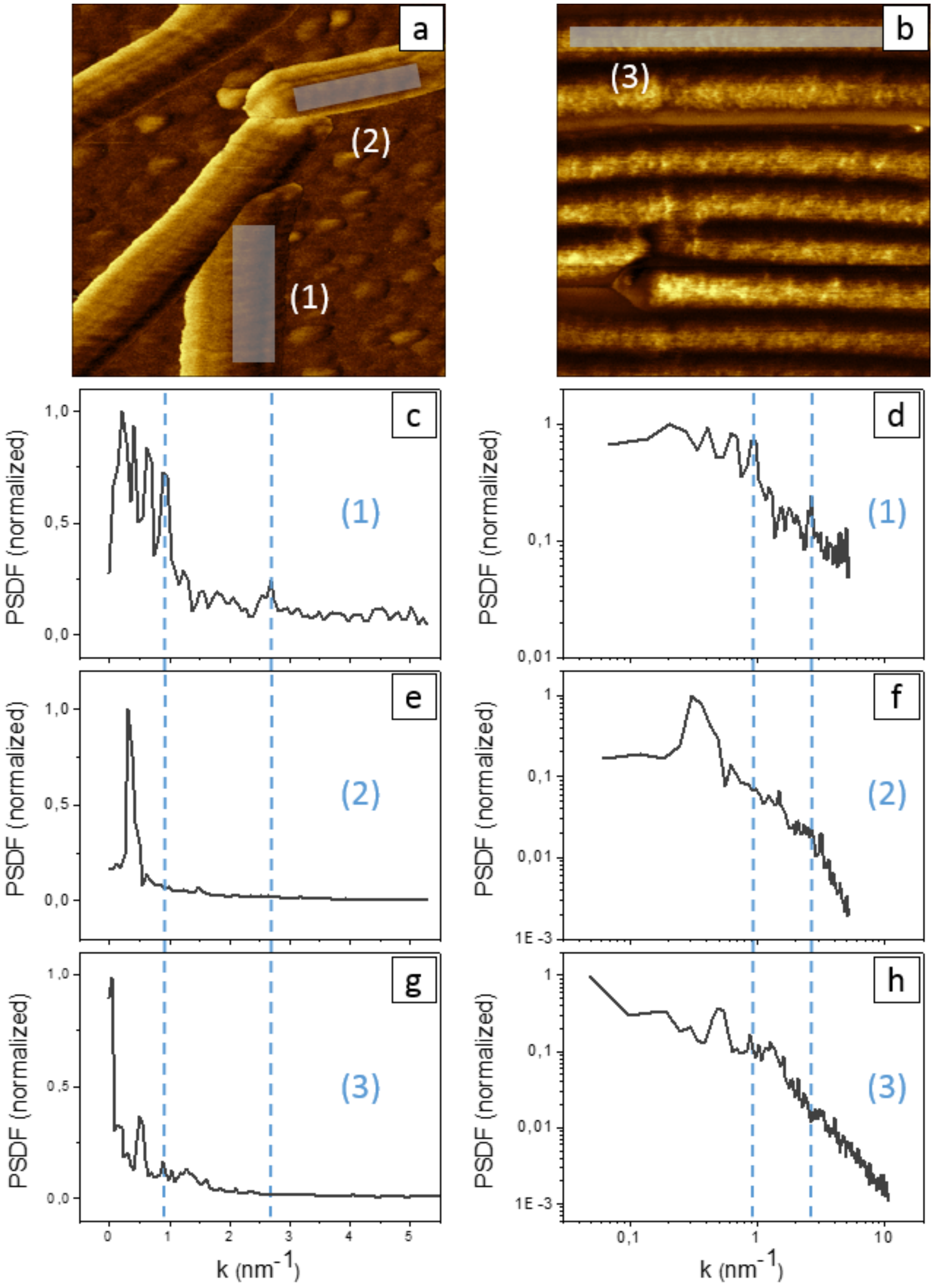

Fig. S3. TMV periodicities. (a, b) Multifrequency phase at upper frequency, φ_2 (a), and standard phase φ_1 (b) AFM images, where the area selected for PSDF analysis is indicated for each virus. (c-h) Corresponding averaged PSDF in linear (c, e, g) and in double logarithmic (d, f, h) scale where the two periodicities of k = 0.91 nm^{-1} (l = 6.9 nm) and k = 2.73 nm^{-1} (l = 2.3 nm) are indicated with dashed vertical lines. Interestingly, the spectrum in (f) shows a correlation length (intersect between two approximately linearly decaying regions) of 2.3 nm.

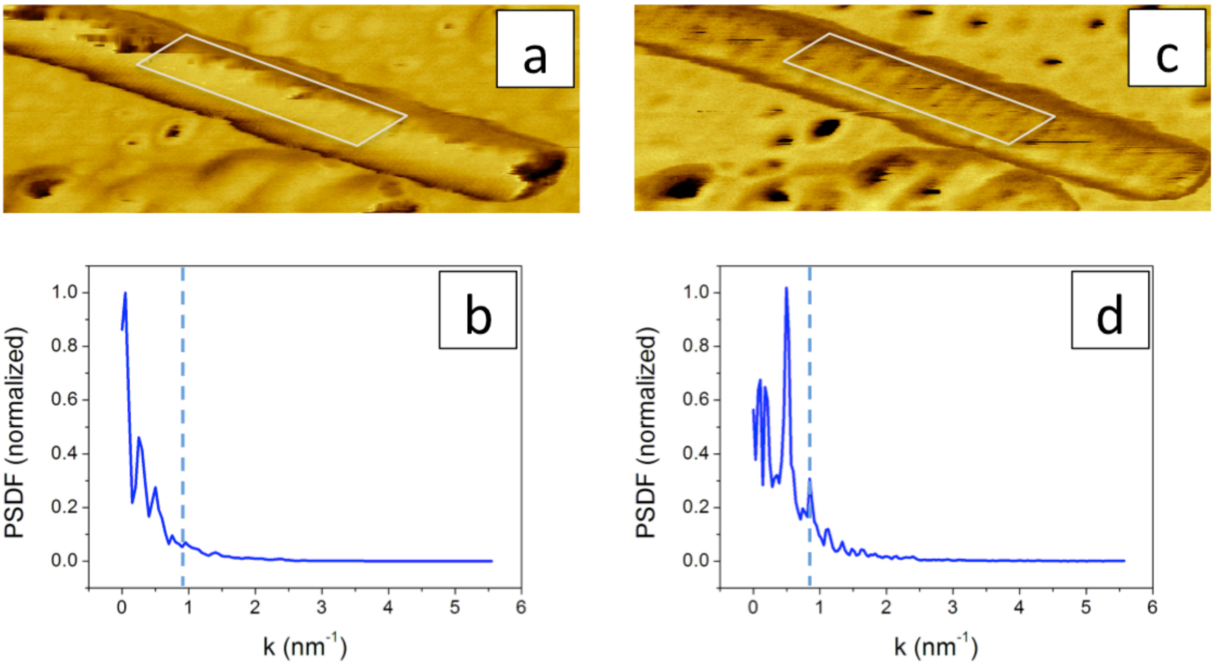

Fig. S4. Comparison of corresponding phase channels from the first and the second mode. (a, c) φ_1 (a) and φ_2 (c) images of a tilted virion. Z scale of the image in (a) is 90-125°. (b, d) PSFD from the region indicated in the images reported in linear scale. The main periodicities in (d) fall at k = 0.49 nm^{-1} (l = 12.7 nm) and k = 0.84 nm^{-1} (l = 7.5 nm).

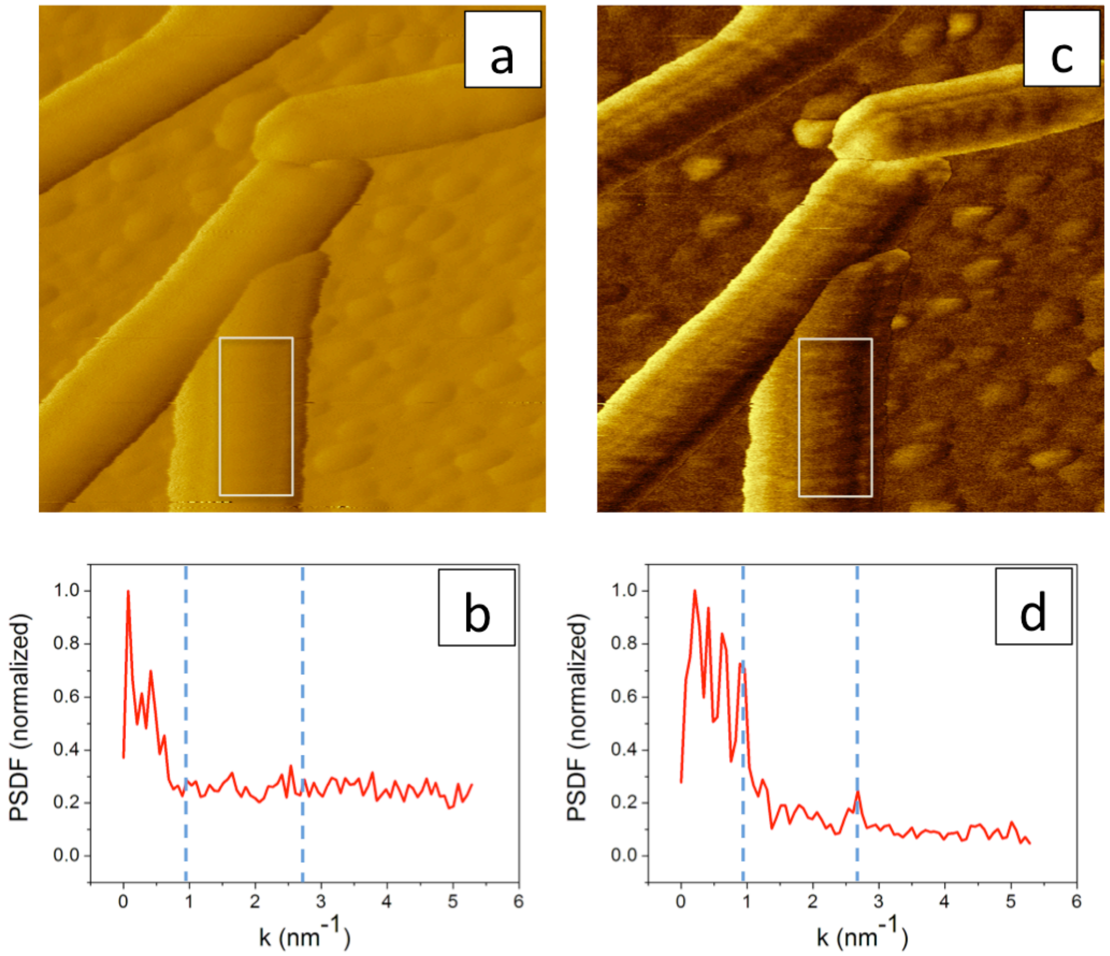

Fig. S5. Comparison of corresponding channels for the first and the second mode. (a, c) A_1 (a) and A_2 (c) images of virions. Z scale of (a) is 0-6 nm. (b, d) PSFD from the region inside the virion reported in linear scale. Indicated are the 6.9 nm and the 2.3 nm periodicities.

References

1. Sader, J. E., Jarvis, S. P. Accurate formulas for interaction force and energy in frequency modulation force spectroscopy. *Appl. Phys. Lett.* **84,** 1801-1803 (2004).
2. Katan, A. J., van Es, M. H., Oosterkamp. T. H. Quantitative force versus distance measurements in amplitude modulation AFM: a novel force inversion technique. *Nanotechnology* **20,** 65703 (2009).
3. Santos, S., Amadei, C. A., Verdaguer, A., Chiesa, M. Size dependent transitions in nanoscale dissipation. *J. Phys. Chem. C*, **117,** 10615-10622 (2013).
